# Successful Outcome of Transfixation Technique with Linen in Dorsal Wrist Ganglion: A Descriptive Cross-sectional Study

**DOI:** 10.31729/jnma.6574

**Published:** 2021-11-30

**Authors:** Pralhad Thapa, Rajesh Bahadur Lakhey

**Affiliations:** 1 Department of Orthopedics and Trauma Surgery, Tribhuvan University Teaching Hospital, Institute of Medicine Maharajgunj, Kathmandu, Nepal

**Keywords:** *ganglion cyst*, *treatment*, *minimally invasive*

## Abstract

**Introduction:**

Ganglion is a commonly encountered soft tissue tumor. Most commonly patients seek treatment for cosmetic issues. There are a variety of treatment options available but very few are effective and cosmetically acceptable. Common treatment techniques have either high recurrence rates or have complications. Treatment by transfixation with linen suture under local anaesthesia on an outpatient basis is an upcoming treatment. The objective of the study is to find out the prevalence of successful outcome of transfixation techniques in treatment of dorsal wrist ganglion.

**Methods:**

This study is a descriptive cross-sectional study undertaken at a tertiary care center from November 2018 to May 2020. Ethical approval was taken from the same institution. Convenient sampling method was used. Patients presenting with dorsal wrist ganglion and consenting for the transfixation underwent the procedure. All the patients were operated under local anesthesia. Statistical analysis was done using Statistical Package for Social Sciences version 24.0. Point estimate was done at 95% Confidence Interval and frequency and percentage were calculated.

**Results:**

Out of 30 cases, 29 (96.66%) have got successful outcomes with transfixation techniques. Among 30 patients, recurrences were seen in 2 (6.66%) cases. Patient satisfaction was high in 27 (90 %) cases. No complication was encountered.

**Conclusions:**

Transfixation technique had good results in terms of low recurrence rate, percentage resolution in size and high patient satisfaction.

## INTRODUCTION

Ganglion cysts are benign soft tissue tumors, most commonly encountered in the wrist, but may occur in any joint. Sixty to seventy percent of ganglion cysts are found in dorsal aspect of wrist & communicate with joint via a pedicle.^12^

There are variety of modalities for treatment of ganglion cysts such as observation, aspiration, intralesional steroid injection, sclerotherapy, transfixation, arthroscopic resection, or surgical excision, but no one of these has been the standard treatment.^3^ Surgical excision remains gold standard for treatment of symptomatic ganglion, but has complications.^4,5^ Transfixation with linen is a newer technique, which have shown promising result and can be carried out under local anesthesia as a day care procedure.^6^

The objective of the study is to find out the prevalence of successful outcome of transfixation techniques in treatment of dorsal wrist ganglion.

## METHODS

The study was a descriptive cross-sectional study conducted in the Department of Orthopedics and Trauma Surgery, Tribhuvan University Teaching Hospital, Institute of Medicine, Maharajgunj, Kathmandu from November 2018 to May 2020. Study was started after getting clearance from the Institutional Review Committee. All patients presenting with ganglion over the dorsum of wrist were included in the study. Volar ganglion, infected ganglion cyst, ganglions less than 5 mm in size, patients with known immune compromised status (diabetes, Human Immunodeficiency Virus (HIV), etc) and patients with local skin lesions (eczema, tinea etc) were excluded from the study. Convenient sampling was done and the sample size was calculated as,

n = Z^2^ × p × q / e^2^

  = (1.96)^2^ × 0.95 × (1-0.95) / (0.08)^2^

  = 28.512

Where,

n= required sample size
Z= 1.96 at 95% Confidence Interval (CI)
p= prevalence of successful outcome of transfixation techniques in treatment of dorsal wrist ganglion, 95% ^7^
q= 1-p
e= margin of error, 8%

Pro-forma was filled for each subject separately. Consent was taken from every patient. On the day of the procedure the patient was given 1gram of flucloxacillin orally, 1 hour before the procedure. Patients allergic to penicillin were given a single dose of erythromycin 500mg. Preoperatively, the skin surface over the ganglion was painted with betadine solution and the region infiltrated with a 2% xylocaine injection. An aspiration was performed to confirm that the swelling under consideration is a ganglion. A clear jelly-like fluid was considered to be confirmatory. Following this, No. 2'0 linen thread was passed through the swelling in two planes perpendicular to each other. Firm pressure and gentle message at the center of the ganglion continued until the swelling completely disappeared. The massage resulted in expulsion of the mucinous contents from the ganglion on the skin surface. The thread was removed on the 4^th^ day at the time of 1^st^ dressing. Following the thread removal the patient was seen at 6 months. During the visit, the size of the swelling along with any other complication was assessed.

**Table 1:** Postoperative patient satisfaction score.

Parameter	Visual analogue score
Pain	0=No pain 10=Unbearable pain
Resolution of presenting complaint	0= Complete resolution 10= No resolution
Functional limitation	0= No limitation 10= Complete loss of function

Calculated score= score of parameter (1+2+3)/3.

Overall results were then calculated on the basis of the percentage of resolution in size and the level of patient satisfaction. Reiterative surgery was classified as poor. Accordingly they were classified into 4 categories: Excellent, Good, Fair and Poor (Table 2).^8^

**Table 2:** Evaluation of overall results.

% resolution in size	High patient satisfaction	Moderate patient satisfaction	Low patient satisfaction	Reiterated surgery
100%	Excellent	Excellent	Good	Poor
70-99%	Excellent	Good	Fair	Poor
<70%	Good	Fair	Poor	Poor

Data was entered in Microsoft Excel sheet. IBM Statistical Package for the Social Sciences (SPSS) version 24 was used to analyze the data. Descriptive statistics were presented with frequencies and percentages for categorical variables. Point estimate at 95% Confidence Interval was calculated, with frequency and percentage.

## RESULTS

Out of 30 cases, 29 (96.66%) got successful outcomes with transfixation techniques. Mean age of the patients was 28±10.6 years (Table 3).

**Table 3:** Distribution of the cases according to age (n= 30).

Age groups	n (%)
10-19	9 (30)
20-29	10 (33.33)
30-39	8 (26.66)
40 or more	3 (10)

Twenty-four (80%) were females and 6 (20%) were males. Dominant hands were involved in 20 (66.66%) whereas ganglion in nondominant hands were found in 10 (33.33%). Left side was involved in 14 (46.66%) and the right side in 16 (53.33%) cases (Table 4).

**Table 4:** Chief complaints of the patients (n= 30).

Chief complaints	n (%)
Cosmesis	9 (30)
Cosmesis and fear of malignancy	5 (16.66)
Cosmesis and pain	16 (53.33)

The time of presentation was less than 1 year in 9 (30%) cases, 1-2 years in 17 (56.66%) cases and 2-4 years in 4 (13.33%) cases.

Range of length of ganglion varied from 1 to 4 cm with mean being 2.3±0.6. Range of breadth of ganglion varied from 1.2 to 3.6cm with mean being 2.3±0.5. Maximum size was 14.04cm^2^ and minimum size being 1.5cm^2^ with mean being 5.5+/-2.9cm^2^.

Recurrence was present in 2 (6.66%) cases. Recurrences were in dominant hands.

Patient satisfaction score at 6 months of follow up was high in 27 (90%) and moderate in 2 (6.66%), poor in 1(3.3%) cases. Overall results were excellent in 27 (90%) and good in 2 (6.66%), poor in 1 (3.33%) cases (Table 5).

**Table 5:** Distribution of the successful results according to age.

Age	Excellent Outcome n (%)	Good outcome n (%)
10-19	8 (26.66)	1 (3.33)
20-29	10(33.33)	0(0)
30-39	7 (23.33)	1 (3.33)
40 and above	2 (6.66)	0(0)

## DISCUSSION

Ganglion cyst in dorsum of hand is the commonly encountered benign soft tissue lump constituting approximately 50 to 70% of swelling of hand and wrist.^9,10^ Different modalities of treatment have been used in the treatment of ganglion cyst ranging from historically hitting with book/bible,aspiration alone, aspirations and injection of corticosteroid with or without hyaluronidase,injection of sclerosing agent, transfixation with suture, radical surgical excision, arthroscopic excision or simply reassurance to the patient.^2,8,11-13^ Despite several options in treating ganglion cyst, recurrence is the main problem encountered. Success rate of various modalities varies considerably.^2,12^

The recurrence rate of ganglions after surgery is quite high.^14-17^ Radical surgery, where ganglia were excised with underlying portion of joint capsule, has low recurrence but high complication rates like persistent pain due of damage to posterior interosseous nerve, scapholunate dislocation, joint stiffness and decreased grip strength.^18^

Even arthroscopic resection of dorsal wrist ganglion, that has gained some momentum in recent years, has high recurrence rate and complications.^19^

Safer, reliable and minimally invasive out-patient department (OPD) procedure for the management of dorsal wrist ganglion has always been searched for.^7^ We wanted to study the results of treatment of ganglion cyst by relatively new OPD procedure-transfixation with linen.

Transfixation of the ganglion with linen is based on the principle that after the procedure, there is acute inflammation within 24 hours. By the end of 3 days, there is laying down of granulation tissue mainly by activation of fibroblasts present in the wall of ganglion. This leads to fibrosis which is complete by 8-10 days.^7^

The mean age was 28+/-10.6 with age range 10-60 years. The relatively common age group in our study as in other studies^7,20,21^ appears to be related to the more involvement of that age group in both indoor and outdoor activities.

In our study, females were affected more than males. Out of the 29 patients enrolled in the study, 25 were females and remaining 5 were males comprising of 83.3% of females. Females are most commonly enrolled in our study similar to other studies.^7,20,21,22^ It can be due to early visit to doctor for cosmetic issues among females.

Dominant hand was involved in 19 (65.5%) cases whereas non-dominant hand was involved in 10 (34.5%) cases in our study. Involvement of dominant hand in our study like in other studies^7,21^ can be explained by the fact that dominant hand is used in various indoor and outdoor activities.

In our study, the patients presented with concern of cosmesis in 9 (30%) cases, concern of cosmesis and fear of malignancy in 14 (13.33%) and concern of cosmesis and pain in 17 (56.67%) cases.

In a study conducted by Sharma Man Mohan, et al.^20^ reported, 20 cases (55.56%) complained of pain, 6 cases (16.63%) complained of pain with cosmesis and 6 cases (16.67%) complained of cosmesis and 4 cases (11.11%) presented with fear of malignancy among 36 cases. Rishi Singhal, et al.^7^ reported pain in 46.2%, followed by cosmesis in 34.6%. Ajaz Ahmad Shah, et al.^22^ reported swelling was the main complaint in 86 cases (100%) followed by pain and discomfort in 52 (60.46%) cases, cosmetic in 49 (56.98%) casesand apprehension of tumor in 34 (39.53%) cases. Rathod, et al.^7^ reported cosmesis as the main complaint in 20 (50%) cases followed by pain and cosmesis in 15 (37.5%) cases and pain only in 5 cases.

In our study, time of presentation varied from 1.5 months to 4 years since onset. Out of 30 cases, 17 (56.66%) presented within the range of 1 to 2 yrs. Delay in time of presentation was seen in some of our cases as they were initially less concerned about the disease.

The size at the time of presentation varied from 1 cm to 4 cm, mean being 2.3+/-0.6cm, in our study. Rishi Singhal, et al.^7^ reported 1 to 2.5cm size at the time of presentation with mean size of 2.04+/-0.45cm. Similarly Sharma Man Mohan, et al.^20^ also reported size ranging form 0.7cm to 3cm with mean being 1.86 cm.

The technique used by us has several advantages. There was no need of hospitalization and the problems of scar, keloid formation and hypertrophy are completely avoided. In contrast to Gang and Makhlouf et al.^22^ who used silk 2/0, we used linen which is a natural twisted multifilament and highly fibrogenic. For this purpose this makes it one of the most suitable suture materials available. Silk on removal leaves pigmentation in the dermis which is undesirable cosmetically to the fair skinned. No lingering pigmentation was seen with the use of linen.

The thread was removed at 3 weeks by Gang and Makhlouf and reported 10 % infection rate.^22^ Similar study by Rathod, et al.^7^ in which suture was removed after 4wks reported infection in one case at 10^th^ day of dressing. Bhavinder Arora, et al.^10^ in which out of 150 patients, 6 cases reported infection. In our study, suture was removed in 4^th^ day, so the need for repeated dressings was obviated. It also prevented infection. None of the patients presented with infection and pigmentation.

In our study, 2 (6.66%) cases had recurrence. Recurrences were seen in the dominant hands. Sharma Man Mohan, et al.^20^ reported recurrence in 2 (2.56%) cases with transfixation with linen. Rathod, et al.^7^ reported a recurrence in one (2.56%) case using transfixation technique with silk suture.

In our study, the patient satisfaction score at follow-up was high in 27 (90%) and moderate in 2 cases (6.66%). Overall results were excellent in 27 (90%) cases and good in 2 (6.66%) cases in the transfixation group. In a study conducted by Rishi Singhal, et al.^8^ high or moderate satisfaction were obtained in 24 patients (92.3%). An overall success rate (Excellent and Good) of 76.9% was achieved with transfixation of ganglion with linen threads.

There were no complications encountered such as scar marks, functional limitation, infection etc.

The limitations of our study were that the principal investigator was unblinded which may have led to observer bias. Small sample size and short duration of study and short time for follow-up were other limitations.

## CONCLUSIONS

Transfixation with linen for the treatment of ganglion cyst had low recurrence rate and high patient satisfaction. Transfixation technique with linen is a minimally invasive procedure, which was found to be effective and cosmetically acceptable in treatment of dorsal wrist ganglion. Our findings were similar to other studies on transfixation with linen for ganglion. We recommend the use of this technique as an outpatient procedure in our setup too.
